# Supplementary figures and images for: Robust expansion of phylogeny for fast-growing genome sequence data
Source: PLoS Comput Biol. 2024 Feb 8;20(2):e1011871. doi: 10.1371/journal.pcbi.1011871 (PMC10898724; doi:10.1371/journal.pcbi.1011871)

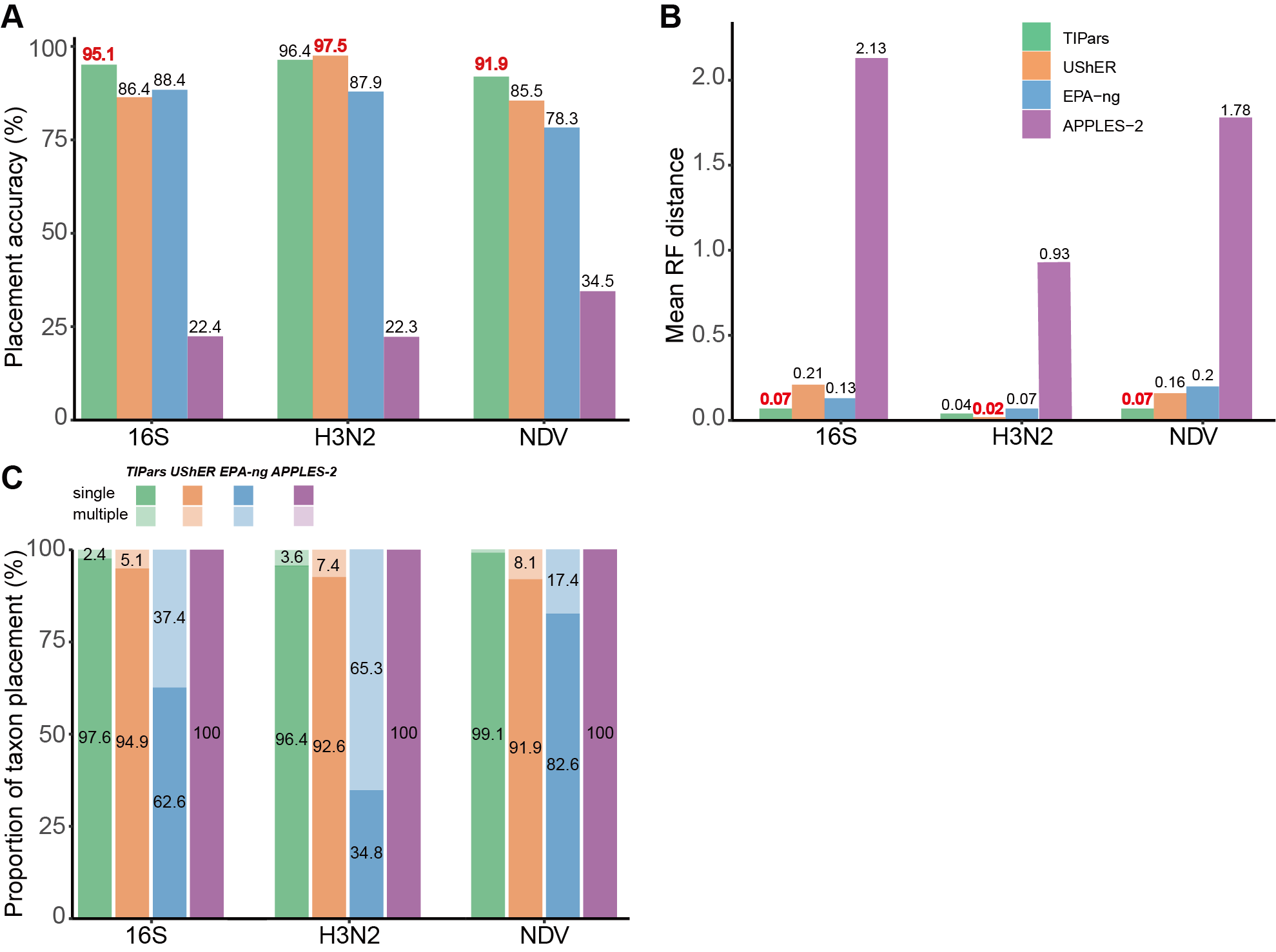

Supplement: S1 Fig — All taxa were removed individually and used for the placement test for TIPars, UShER, EPA-ng and APPLES-2 in 16S, H3N2 and NDV datasets. Note that the ancestral sequences of TIPars and mutation-annotated tree of UShER have not been reconstructed for each leave-one-out test due to the high computational requirement that would cause a bias for their accuracies. RAPPAS was excluded because of its large computation for the ‘pkDB’ database. (A) Bars represent the placement accuracy on 16S, H3N2 and NDV datasets. The highest accuracy in each dataset is highlighted in red. (B) Bar charts representing the mean RF distance calculated from the single taxon placement results on 16S, H3N2 and NDV datasets. The lowest mean RF distance in each dataset is highlighted in red. Panel A and B share the same figure legend in B. (C) Stacked bar charts showing the proportions of single and multiple placement results on 16S, H3N2 and NDV datasets. Proportions with > 0.1% are indicated within the bars. (TIF) [file pcbi.1011871.s001.tif]

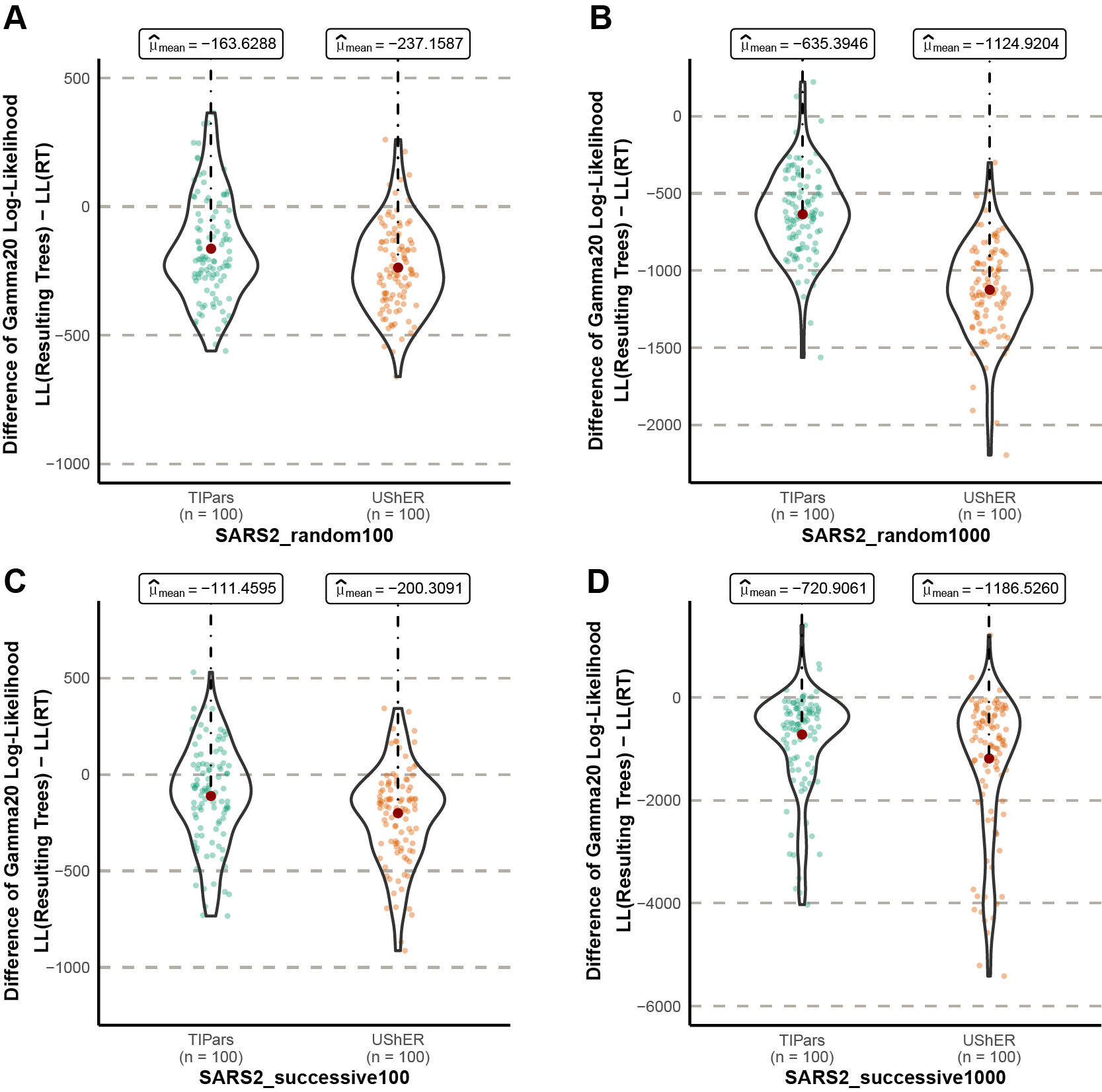

Supplement: S2 Fig — (A) Violin plots show the distribution of paired differences for the 100 sets of random 100 multiple sequence insertions. (B) Violin plots show the distribution of paired differences for the 100 sets of random 1000 multiple sequence insertions. (C) Violin plots show the distribution of paired differences for the 100 sets of successive 100 multiple sequence insertions. (D) Violin plots show the distribution of paired differences for the 100 sets of successive 1000 multiple sequence insertions. (TIF) [file pcbi.1011871.s002.tif]

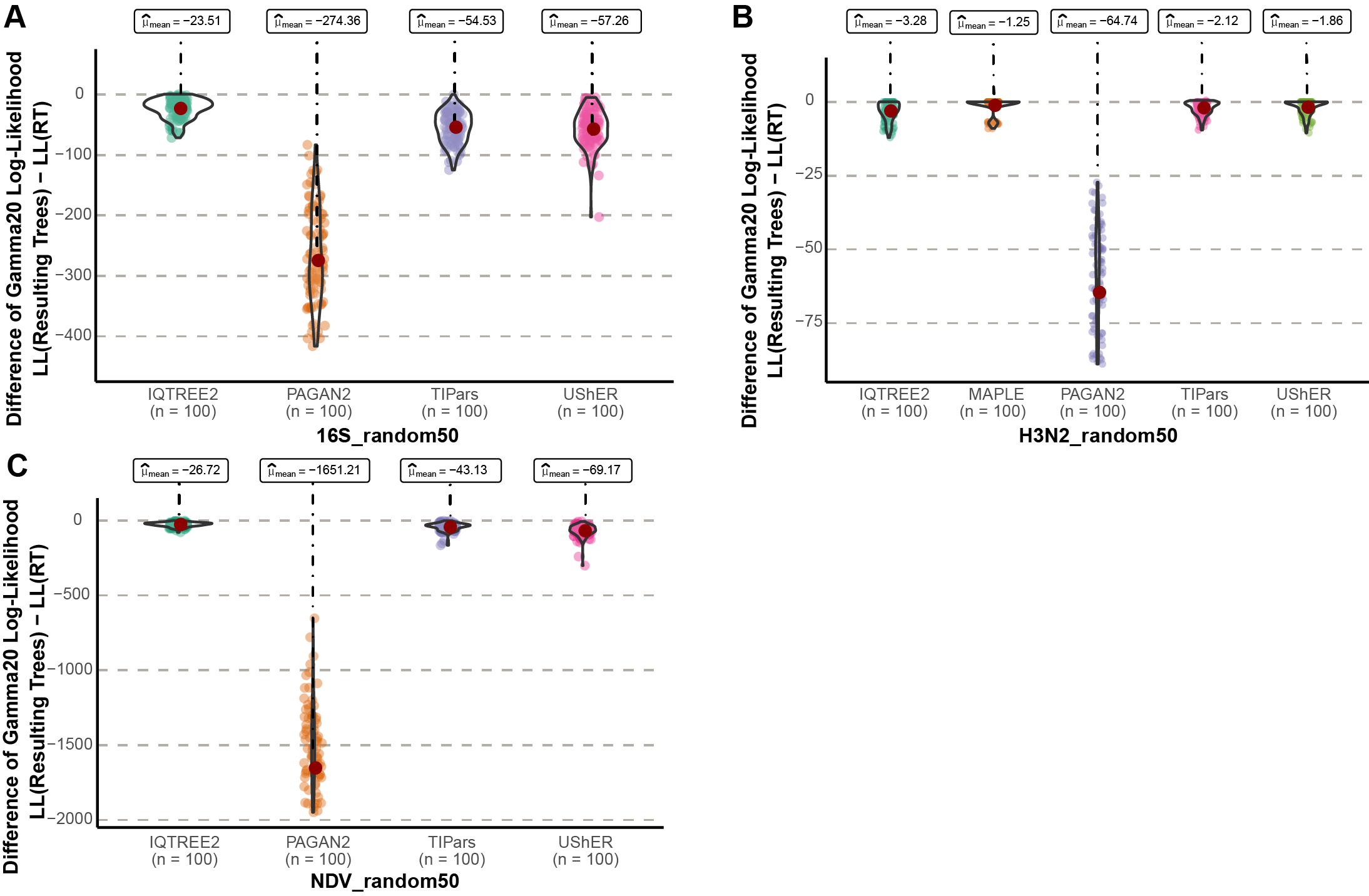

Supplement: S3 Fig — (A) Violin graphs show the distribution of the paired differences for 100 sets of 50 random multiple sequence insertions in 16S dataset. The mean difference of MAPLE was 10837.68. (B) Violin graphs show the distribution of the paired differences for 100 sets of 50 random multiple sequence insertions in the H3N2 dataset. (C) Violin graphs show the distribution of the paired differences for 100 sets of 50 random multiple sequence insertions in the NDV dataset. The mean difference of MAPLE was 41811.12. (TIF) [file pcbi.1011871.s003.tif]

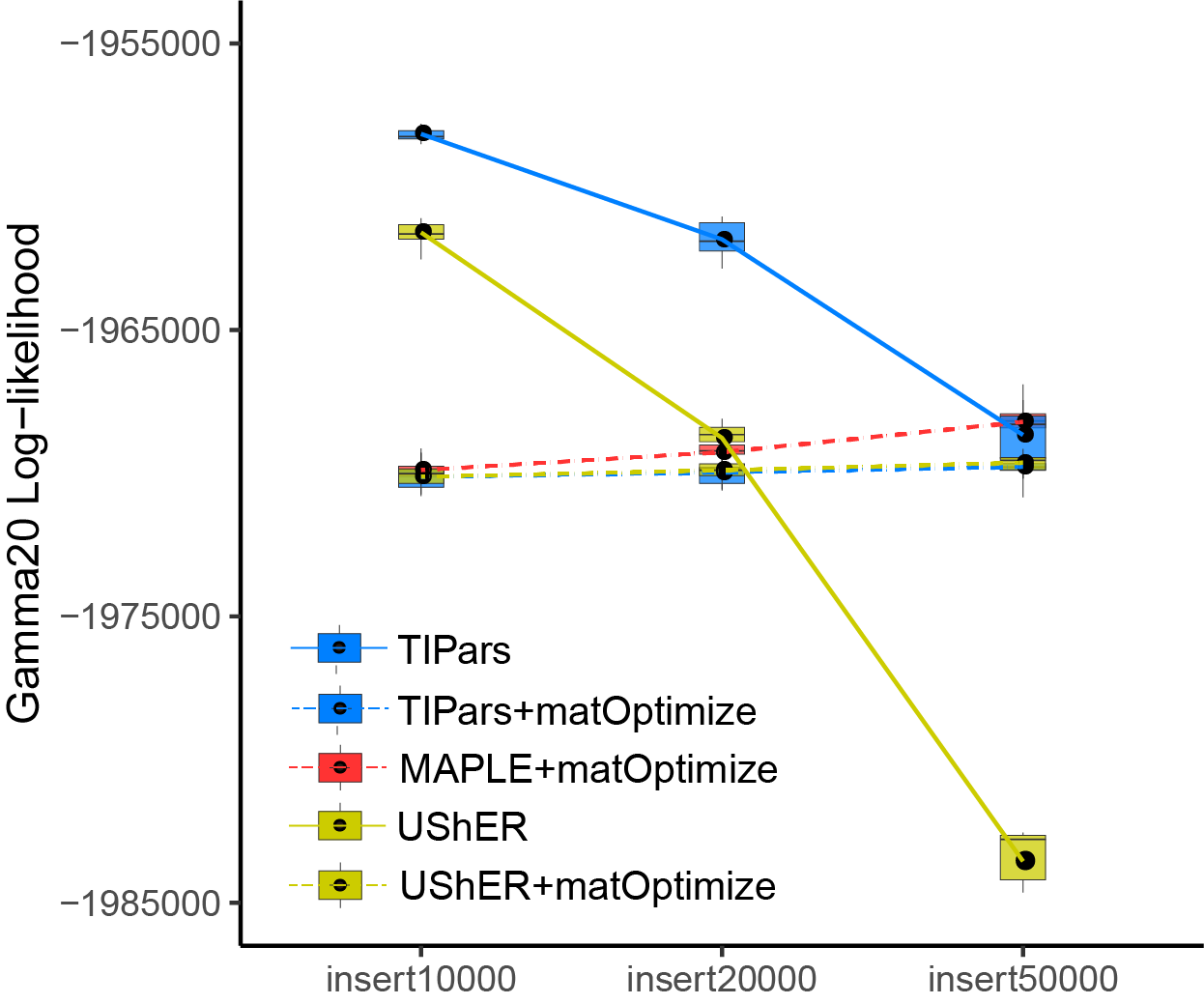

Supplement: S4 Fig — Larger values represent more likely estimates. ‘X+matOptimize’ indicates the refined trees of X with tree topology optimized by matOptimize. (TIF) [file pcbi.1011871.s004.tif]

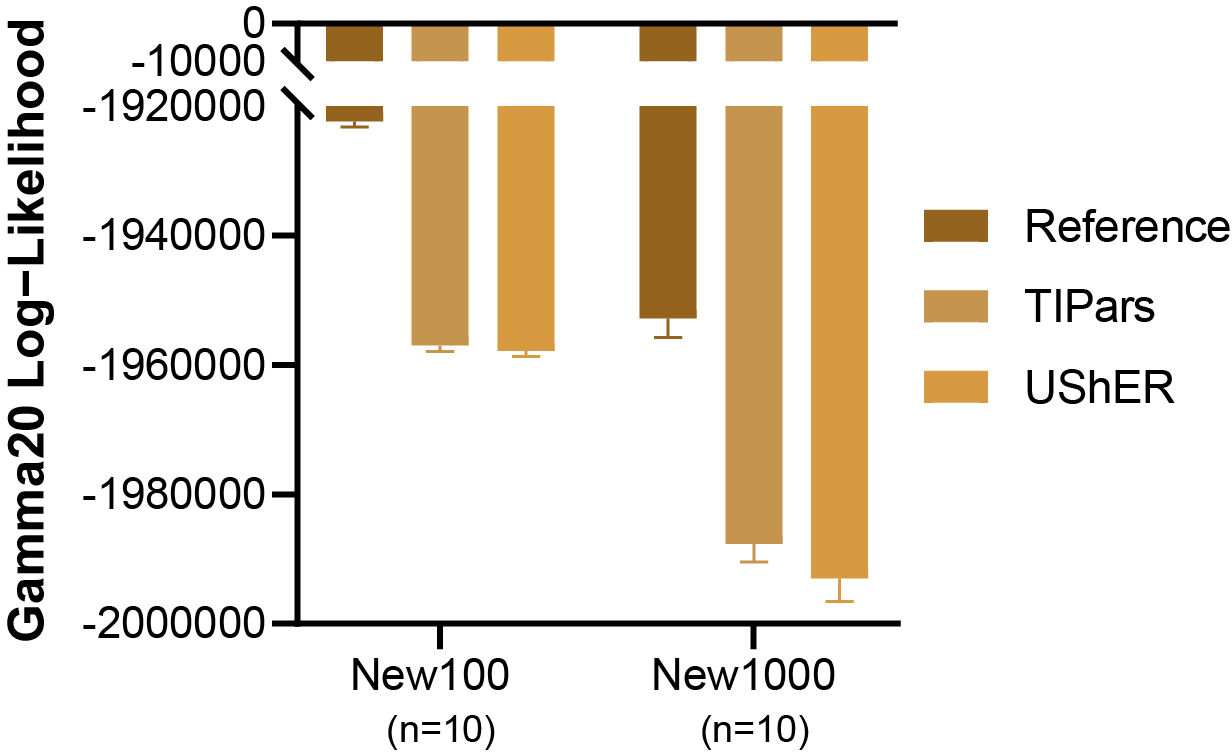

Supplement: S5 Fig — The reference tree was constructed by FastTree v2.1.11 (double-precision version) using the alignment of the newest added sequences and the sequences in SARS2-100k dataset. (TIF) [file pcbi.1011871.s005.tif]
