# Supplementary material for: Robust expansion of phylogeny for fast-growing genome sequence data
Source: PLoS Comput Biol. 2024 Feb 8;20(2):e1011871. doi: 10.1371/journal.pcbi.1011871 (PMC10898724; doi:10.1371/journal.pcbi.1011871)
Supplement: Attachment — Submitted filename: Reply_to_ReviewComments_R1.docx [file pcbi.1011871.s022.docx]

**Authors’ Reply to Review Comments**

The reviewers all agreed that this study is meritorious, and I agree. Each reviewer raised important points that must be addressed in a revision. Further, I agree with two of the reviewers that a comparison against MAPLE would greatly benefit this manuscript.

Reviewer's Responses to Questions

**Comments to the Authors:**

**Please note here if the review is uploaded as an attachment.**

Reviewer #1: This work (TIPars) integrates parsimony analysis with pre-computed ancestral sequences. It took only 21 seconds to insert 100 genomes into a 100k-taxa reference tree using an efficient 1.4 GB peak memory, outperforming other methods for moderately similar sequences (in terms of accuracy), although second to UShER for highly similar and divergent scenarios. This study is well designed and done in presenting detailed metrics of performance (RF distances, log-likelihood, running time, peak memory usage, etc.) for various datasets and scenarios, including leave-one-out insertion, leave-many-out insertion, and inserting novel sequences. Results are well discussed and interpreted. The manuscript is well written. I only have a number of minor suggestions below.

**Authors’ reply:** Thank you so much for the summary of TIPars

1. It was mentioned in line#98 that “RAPPAS and PAGAN2 were unable to complete within 96 hours, hence, no data were available.” In Table 1 caption, it was mentioned they “could not be run on this dataset”. It’s better to clarify that no success was due to that the job could not be done within 96 hours. Was it running but simply did not get result in time? Or there were factors preventing the program to run anyway?

**Authors’ reply:** The insertion of 100 SARS-CoV-2 genome sequences into a 100k-taxa reference tree using RAPPAS and PAGAN2 could not be done within 96 hours. We have added this statement to Table 1 caption.

2. It would probably be better that Fig 1B has its own chart legends as in Fig 1C and Fig 1D does, although they were texted in the figure legend. Similar concerns are also found in other figures.

**Authors’ reply:** We have rephrased the legend for each panel of all figures and supplementary figures individually.

3. As stated in line#318 to #323, TIPars could be compromised with incorrect placements accumulated as multiple sequences are inserted sequentially. The authors suggest conducting tree refinement for remedy. It will be great if one such example can be included in this manuscript, if possible.

**Authors’ reply:** Thanks for the suggestion. We have added a benchmark to use matOptimize (Ye et al., Bioinformatics 2022) to refine the topology of inserted trees from TIPars in SARS2-100k dataset and the result is described at Lines 252 to 273 of the revised manuscript (Figure 4).

The refined trees of TIPars achieved smaller parsimony scores for leave-out 10000, 20000 and 50000 tests, but smaller tree log-likelihoods (except insert50000) and larger RF distances. A possible reason is that matOptimize applies fast subtree pruning and regrafting (SPR) moves fully based on parsimony criteria without a promise on the tree log-likelihood. While the provided SARS2-100k reference tree was built by a maximum likelihood method (IQTREE2), the RF distance after refinement by matOptimize may not become smaller.

**Figure 4**

**Multiple sequences insertion with tree topology refinement.** (A) RF distances between inferred trees and the reference tree for 10 leave-out tests of 10000, 20000 and 50000 taxa in SARS2-100k dataset respectively. Smaller values correspond to more similar to the reference tree. ‘X+matOptimize’ indicates the refined trees of X with tree topology optimized by matOptimize. (B) Log-likelihoods (LK) (computed by FastTree2 (double-precision version)) of phylogenies inferred by different methods. Larger values represent more likely estimates. (C) Parsimony scores (computed by UShER) of phylogenies inferred by different methods. Smaller values represent smaller number of substitutions needed for each site.

Reviewer #2: In this manuscript, Ye at al. present TIPars, a phylogenetic placement algorithm for the rapid expansion of microbial phylogenies. In particular, according to the authors, TIPars achieves a better balance of efficiency and accuracy relative to prior tools and works well for similar as well as divergent sequences. Supporting evidence has been provided using single and multiple taxa insertion on four different datasets (SARS-CoV-2, Influenza, NDV and 16S). Source code is provided and is also packaged into a web app. While the manuscript is easy to read and aims to solve an important problem, there are certain aspects of the manuscript that need improvement, as I highlight below.

**Authors’ reply:** We thank the reviewer for the positive comments and the useful suggestions detailed below.

First, to my understanding, TIPars uses a very similar parsimony-based placement strategy as UShER. However, it differs in the reconstruction of ancestral sequences, using likelihood instead of parsimony. This seems to help it achieve a better trade-off of efficiency and accuracy. However, a more sophisticated approach, but in a similar vein, was recently developed in MAPLE (De Maio et al., Nature Genetics 2023). In particular, MAPLE uses a “Parsimonious Likelihood” approach that also achieves a good speed-accuracy trade-off, and therefore, a comparison to it is warranted.

**Authors’ reply:** Thanks for the suggestion. We tested MAPLE (v0.3.4) for multiple taxa insertion in 16S, H3N2 and NDV datasets and the result is described at Lines 219 to 228 and Figure 3E to 3H in the revised manuscript (the benchmark on SARS-CoV-2 dataset is presented in the next comment). To make it fair, we ran MAPLE without topology refinement (Table S15) since TIPars and UShER will not alter the backbone tree for phylogeny expansion.

MAPLE outperformed other methods in H3N2 dataset with higher Gamma20 log-likelihoods (LL) of the inserted trees while a significant drop of LLs was observed in more divergent datasets 16S and NDV (Figure 3F to 3H). The RF distance from MAPLE inserted trees is comparatively larger in 16S, H3N2 and NDV datasets that is presented in Table S6. It might be expected that MAPLE is designed for highly similar sequences and may break down in divergent scenario (De Maio et al., Nature Genetics 2023). MAPLE implemented a local phylogenetic placement algorithm where the ‘best’ placement of a query sequence is searched on a subset of branches in the tree while TIPars and UShER are global search by a transversal of all branches. Phylogenetic tree is not a well classified data structure (such as red–black tree), a local search may stop too early to retrieve the global best placement especially for divergent phylogeny which contains long branches.

**Figure 3E-H**

**Multiple sequences insertion performance in 16S, H3N2 and NDV datasets**. (E) Distribution of paired differences in the RF distances between the resulting trees generated by TIPars and UShER (TIPars - UShER) on 16S, H3N2 and NDV random 50 multiple sequences insertions. (F) Distribution of the paired differences in the Gamma20 log-likelihoods between the resulting trees generated by TIPars and the four other programs (TIPars - Others) on 16S random 50 multiple sequences insertions. (G) Distribution of the paired differences in the Gamma20 log-likelihoods between the resulting trees generated by TIPars and the four other programs (TIPars - Others) on H3N2 random 50 multiple sequences insertions. (H) Distribution of the paired differences in the Gamma20 log-likelihoods between the resulting trees generated by TIPars and the four other programs (TIPars - Others) on NDV random 50 multiple sequences insertions. P-values for the right-sided paired t-tests are indicated by the asterisk on top of each violin diagram, where p<0.05 is indicated by one pink asterisk (*), p<0.01 by two orange asterisks (**) and p<0.001 by three red asterisks (***).

Second, while the authors talk about TIPars’ application to phylogeny expansion, I don’t think this ability has been convincingly demonstrated. Importantly, greedy placement strategies are known to accumulate suboptimalities during phylogenetic expansion. This issue is ameliorated in SARS-CoV-2 UShER trees by using matOptimize (Ye et al., Bioinformatics 2022). MAPLE also uses SPR moves to optimize the topology periodically. Though the authors have experimented with multiple taxa insertion, the number of taxa inserted is small relative to the size of the starting phylogeny, and it is unclear if TIPars would be able to accurately maintain the expanding phylogeny. The scalability of TIPars’ approach would likely also be limited by tby the methods to build the MSA and the likelihood-based reference tree. I would like to see the authors address this important issue in their revision.

**Authors’ reply:** We agree with the limitation of our original benchmark for a relatively small number of query sequences (100 and 1000). We have conducted a relatively large insertion benchmark in SARS2-100k dataset for 10 leave-out tests of 10000 (insert10000), 20000 (insert20000) and 50000 taxa (insert50000) respectively (Figure 4) and the result is described at Lines 252 to 273 of the revised manuscript. In this benchmark, we compared tree log-likelihood (LK) calculated by FastTree2 (double-precision version) instead of Gamma20 Log-likelihood (LL) because it returns ‘-nan’ for LLs of inserted trees from MAPLE. But we present the LL results for TIPars and UShER in Figure S4.

TIPars achieved smallest RF distance and highest log-likelihoods (LK) of inserted trees (Figure 4A and 4B). UShER had the smallest tree parsimony score in insert20000 and insert50000 tests while MAPLE had the smallest parsimony score in insert10000 but an increased parsimony score was observed when inserting more queries (Figure 4C). For the computational performance, MAPLE took 33 seconds to insert 100 genomes into a 100k-taxa reference tree using 24.04 GB peak memory while those of TIPars were 21 seconds and 1.39 GB (Table 1).

The phylogenetic placement algorithms implemented in TIPars, UShER and MAPLE do not alter the backbone tree topology. matOptimize (Ye et al., Bioinformatics 2022) and MAPLE developed SPR moves to improve the inserted trees. Hence, we utilized matOptimize to refine the topology of inserted trees from TIPars, UShER and MAPLE. We did not apply the SPR moves in MAPLE because of its high computation cost (it is a single-thread program and took 60.85 hours to refine a 100k-taxa tree). We confirmed with the author of MAPLE that the efficiency of MAPLE would be influenced by the number of gaps in the multiple sequence alignment (MSA) of taxa. In our SARS2-100k dataset, insertions to the reference genome (hCoV-19/Wuhan/WIV04/2019/EPI_ISL_402124) were included in the MSA while those in MAPLE (De Maio et al., Nature Genetics 2023) are not.

After topology refinement, the refined trees had significantly smaller parsimony scores but larger RF distance for trees of TIPars and UShER (Figure 4A and 4C). The inserted trees from TIPars (without topology refinement) had larger LKs than those of being refined in insert10000 and insert20000 and comparatively similar LK values were observed in insert50000 (Figure 4B). A possible reason is that matOptimize is a fully parsimony-based method without a promise on the tree log-likelihood. Our provided reference tree in SARS2-100k dataset was built by a maximum likelihood method (IQTREE2), so the RF distance after refinement by matOptimize may not become smaller. The refined trees from MAPLE had smaller RF distances, larger LKs and smaller parsimony scores compared to those of without refinement which suggests a tree refinement can be useful for the inserted tree of MAPLE.

An MSA (including taxa in backbone tree and query sequences) and a reference tree are both required to TIPars, UShER and MAPLE for phylogeny expansion. But we acknowledge that when doing a tree topology refinement after adding query sequences, the ancestral sequences to TIPars should be reconstructed again which will take additional computation cost. And we have added this statement to the discussion session at Lines 355-359 of the revised manuscript.

**Figure 4**

**Multiple sequences insertion with tree topology refinement.** (A) RF distances between inferred trees and the reference tree for 10 leave-out tests of 10000, 20000 and 50000 taxa in SARS2-100k dataset respectively. Smaller values correspond to more similar to the reference tree. ‘X+matOptimize’ indicates the refined trees of X with tree topology optimized by matOptimize. (B) Log-likelihoods (LK) (computed by FastTree2 (double-precision version)) of phylogenies inferred by different methods. Larger values represent more likely estimates. (C) Parsimony scores (computed by UShER) of phylogenies inferred by different methods. Smaller values represent smaller number of substitutions needed for each site.

Lastly, the authors are probably not using the latest versions of the baseline tools. For example, they used v0.3.8 for UShER. UShER v0.6 provides additional optimizations and includes usher-sampled, which is over 10x faster while guaranteeing the same placements. It’s also not clear why TIPars is ~10x slower and ~2x less memory efficient than UShER though its placement strategy is similar. I hope the authors can expand on this.

**Authors’ reply:** We have re-tested the speed and peak memory usage of UShER v0.6.2 for inserting 100 SARS-CoV-2 genome samples into SARS2-100k reference tree (Table 1) and inserting 50 samples into the 16S 800-taxa reference tree (Table S1). The runtime and peak memory usage are close to the old ones.

For the memory usage, when inputs are Newick format reference tree and VCF format MSA, TIPars stores the tree using BEAST library and MSA as a hashmap. UShER uses its mutation-annotated tree (MAT) data structure to store the tree and MSA which is more memory efficient. For the speed comparison, the parallelization implemented in TIPars is using the Java parallelstream while UShER depends on the C++ library ‘oneTBB’. Due to different parallel libraries and programming languages, the speed may not be easy to judge without further technical investigation.

A few minor suggestions and questions for the authors:
1. How did the authors obtain the PANGO lineage assignments for the SARS-CoV-2 sequences? To my understanding, the PANGO system has switched to UShER (called PUSHER) for lineage assignments.

**Authors’ reply:** PANGO lineage labels were extracted from the metadata downloaded from GISAID on July 6, 2021. We have added this description at Lines 407-408 of the revised manuscript.

2. What were the runtimes required to generate the MSAs, reference trees and the ancestral sequences for the different datasets? How frequently does this need to be done to maintain accuracy?

**Authors’ reply:** We have added the runtimes and memory usages to generate MSAs, reference trees and ancestral sequences for 16S, H3N2 and NDV dataset in Table S13. For SARS2-100k dataset, the MSA is downloaded from GISAID; the tree was built by IQTREE2 using around 14 hours with 40 Intel Xeon Gold 6242 CPU cores (added at Lines 412-413 of the revised manuscript) and the ancestral sequences was constructed by PastML using 4 hours (Table S2).

**S13 Table. Runtime and memory usage for 16S, H3N2 and NDV dataset preparation**.

| Runtime (mm:ss.f) /  Peak memory usage (GB) | Alignment  (MUSCLE) | Tree construction (RAxML) | Ancestral sequence reconstruction (Hyphy) | |
| --- | --- | --- | --- | --- |
| 16S | 13:03.3 / 2.38 | 15:39.2 / 0.29 | 09:33.3/0.145 |  |
| H3N2 | 4:42.4 / 0.13 | 11:39.2 / 0.89 | 01:27.1/0.03 |  |
| NDV | 36:26.4 / 9.3 | 12:52.6 / 0.98 | 22:09.5/0.312 |  |

Tests were run on a server with 32 Intel Xeon Gold 6242 CPU cores.

Figure 4 shows the accuracy performance of TIPars when expanding the tree to 111% (insert10000), 125% (insert20000) and 200% (insert50000) with and without tree topology refinement. Users could determine the frequency to do a topology refinement according to their needs. After a tree topology refinement, the ancestral sequences should be reconstructed again for TIPars. We have added this statement to the discussion session at Lines 355-359 of the revised manuscript.

3. Is there a limit to the tree size that TIPars can handle? Why not evaluate the performance on millions of SARS-CoV-2 sequences that are available?

**Authors’ reply:** Based on our test, TIPars took 21 seconds to insert 100 genomes into a 100k-taxa reference tree using an efficient 1.4 GB peak memory (Table 1). When considering the input tree size for TIPars to handle, the bottleneck is how to infer a high-quality reference tree. For example, IQTREE2 and Fasttree2 both required over 100GB for a 100k-taxa reference tree construction.

The available huge trees with millions of SARS-CoV-2 taxa are built by UShER in GISAID and Genome Browser. There is a potential bias to the performance of UShER if using these trees.

4. Does PastML use maximum parsimony (line 373)? I thought the goal was to infer ancestral sequences using likelihood.

**Authors’ reply:** PastML provides both maximum parsimony and maximum likelihood methods for ancestral sequence reconstruction. TIPars is agnostic to how the ancestral sequences are computed, and users are able to fine tune the method on their needs.

In our benchmarks, we used the maximum likelihood method to construct ancestral sequences for 16S, H3N2 and NDV datasets while using maximum parsimony method for SARS-CoV-2 datasets to reduce the computation cost.

5. Why have the authors used different methodologies for generating reference trees of different datasets (IQ-Tree GTR for one and RaxML GTG+G in the remaining)?

**Authors’ reply:** We used RAxML (GTR+G) to build reference trees for 16S, H3N2 and NDV datasets. But for the SARS2-100k dataset, RAxML could not be done within 1 week so we turn to IQTREE2 (GTR). And based on our test on the SARS2-100k dataset, when using GTR+G mode, IQTREE2 would stop and report ‘numerical underflow’ error.

6. In addition to RF distance, it might be helpful to also evaluate the distance from the correct node.

**Authors’ reply:** RF distance is used in both UShER and MAPLE papers, so we also used RF distance. In addition to true positive accuracy (Figure 1B), we provide the mean RF distance comparison in Figure 1C, Figure 3 and Figure 4 to evaluate the distance to the correct node which represents the different partitions of the resulting tree due to the query insertion. When considering the branch length as well as tree topology, we used the tree loglikelihood as a gold standard for comparisons (Figure 3-5).

7. Typo: “verified a superiority” -> “verify the superiority”

**Authors’ reply:** We revised it at Line 320 of the revised manuscript.

Reviewer #3: In their Manuscript Ye et al. describe a novel method for placing taxa on an existing phylogenetic tree. Similar 'online' phylogenetic approaches have proven very useful for analyses of large, growing datasets since they avoid the computational burden of reestimating large phylogenies from scratch each time new data becomes available. Existing approaches typically use maximum likelihood, minimum evolution or maximum parsimony to determine where a new sequence falls on an existing tree. As the the authors discuss, there is a trade-off between accuracy and speed, with full likelihood methods requiring longer run times to reach more accurate reconstructions. The proposed method, TIPars, provides a flexible compromise between the accuracy of maximin likelihood and the speed of parsimony.

TIPars evaluates taxa placement by estimating the number of substitutions between a query taxa and the precomputed ancestral sequence present on each branch. Because TIPars is agnostic to how these ancestral sequences are computed, the user is able fine tune the method to their needs. More accurate, slower results may be achieved if the ancestral states are estimated with maximum likelihood. However, this step is only needed once. Because placement is based on a simple parsimony-like score it remains efficient.

The author rigorously benchmark TIPars against standard tools with an array of datasets that vary in size and diversity. The tool is available as a command line interface as well as a well a web service, which should make it easy to use and share results. I have only the following concerns with the manuscript.

**Authors’ reply:** We thank the reviewer for his in-depth summary of TIPars and its significance. We have replied to the comments point by point as follows.

The authors show that TIPars outperforms USHER in a number of settings. One of these, is in SARS-CoV-2 datasets where both USHER and TIPars use parsimony reconstructions. It is unclear why TIPars does better, when similar methods are used. The authors suggest indels may be informing the taxa placement, but the methods don't mention how indels are used, and it seems like the MSA was generated by aligning to a reference.

**Authors’ reply:** Based on our understanding on UShER, nucleotide insertions to the reference genome (hCoV-19/Wuhan/WIV04/2019/EPI_ISL_402124) were excluded in the MSA while those in TIPars are included.

Possibly related to the point above, it would be good of the authors to provide some of the reasoning behind their tie-breaking criteria. Are these criteria better suited for certain datasets like densely sampled outbreaks?

**Authors’ reply:** The first priority is to select the branch with node A containing the most numbers of child nodes that is to place the query at a larger subtree. The second priority is to select the branch with node A of the lowest node height (the total branch length on the longest path from the node to a leaf) that is to place the query at an ancestor with less accumulated mutations. We have added this description at Lines 380-383 of the revised manuscript. Based on our results, these criteria are practical for similar (SARS-CoV-2 and H3N2) and divergent (NDV and 16S) phylogenies.

I hesitate to suggest more tools to benchmark against, but it would be informative to include the approximate likelihood used in MAPLE (De Maio, 2023). This approximation may break down in more divergent datasets, but has been shown to perform well for large, low-diversity datasets.

**Authors’ reply:** Thanks for the suggestion. We tested MAPLE (v0.3.4) for multiple taxa insertion in 16S, H3N2, NDV (Figure 3E to 3H) and SARS2-100k datasets (Figure 4). To make it fair, we ran MAPLE without topology refinement (Table S15) since TIPars and UShER will not alter the backbone tree for phylogeny expansion. In the benchmark on SARS2-100k dataset, we compared tree log-likelihood (LK) calculated by FastTree2 (double-precision version) instead of Gamma20 Log-likelihood (LL) because it returns ‘-nan’ for LLs on inserted trees from MAPLE. But we present the LL results for TIPars and UShER in Figure S4. Results are described at Lines 219-228 and 252-273 of the revised manuscript.

MAPLE outperformed other methods in H3N2 dataset with higher Gamma20 log-likelihoods (LL) of the inserted trees while a significant drop of LLs was observed in more divergent datasets 16S and NDV (Figure 3F to 3H) and ran slowest in 16S dataset (Table S1). The RF distance from MAPLE inserted trees is comparatively larger in 16S, H3N2 and NDV datasets (Table S6). It may be expected that MAPLE is designed for highly similar sequences and may break down in divergent scenario (De Maio et al., Nature Genetics 2023).

For leave-out tests of 10000 (insert10000), 20000 (insert20000) and 50000 taxa (insert50000) in SARS2-100k dataset, TIPars achieved smallest RF distance and highest log-likelihoods (LK) of the inserted trees (Figure 4A and 4B). UShER had the smallest tree parsimony score in insert20000 and insert50000 tests while MAPLE had the smallest parsimony score in insert10000 but an increased parsimony score was observed when inserting more queries (Figure 4C). For the computational performance, MAPLE took 33 seconds to insert 100 genomes into a 100k-taxa reference tree using 24.04 GB peak memory while those of TIPars were 21 seconds and 1.39 GB (Table 1).

**Figure 3E-H**

**Multiple sequences insertion performance in 16S, H3N2 and NDV datasets**. (E) Distribution of paired differences in the RF distances between the resulting trees generated by TIPars and UShER (TIPars - UShER) on 16S, H3N2 and NDV random 50 multiple sequences insertions. (F) Distribution of the paired differences in the Gamma20 log-likelihoods between the resulting trees generated by TIPars and the four other programs (TIPars - Others) on 16S random 50 multiple sequences insertions. (G) Distribution of the paired differences in the Gamma20 log-likelihoods between the resulting trees generated by TIPars and the four other programs (TIPars - Others) on H3N2 random 50 multiple sequences insertions. (H) Distribution of the paired differences in the Gamma20 log-likelihoods between the resulting trees generated by TIPars and the four other programs (TIPars - Others) on NDV random 50 multiple sequences insertions. P-values for the right-sided paired t-tests are indicated by the asterisk on top of each violin diagram, where p<0.05 is indicated by one pink asterisk (*), p<0.01 by two orange asterisks (**) and p<0.001 by three red asterisks (***).

**Figure 4**

**Multiple sequences insertion with tree topology refinement.** (A) RF distances between inferred trees and the reference tree for 10 leave-out tests of 10000, 20000 and 50000 taxa in SARS2-100k dataset respectively. Smaller values correspond to more similar to the reference tree. ‘X+matOptimize’ indicates the refined trees of X with tree topology optimized by matOptimize. (B) Log-likelihoods (LK) (computed by FastTree2 (double-precision version)) of phylogenies inferred by different methods. Larger values represent more likely estimates. (C) Parsimony scores (computed by UShER) of phylogenies inferred by different methods. Smaller values represent smaller number of substitutions needed for each site.

The authors should provide more discussion around how TIPars could be used in a large outbreak. The need to build and maintain a large MSA is more intensive than current approaches that align to a reference, and how could users apply the NNI and SPR moves suggested in line 323? (I believe USHER allows for these moves, but using USHER to do so would probably defeat the purpose).

**Authors’ reply:** We agree that it is challenging to build a large MSA including insertions and deletions rather than aligning to a particular reference. The MSA (including taxa in the backbone tree and query sequences) is required to TIPars, UShER and MAPLE for phylogeny expansion. We have added this statement at Lines 359-361 of the revised manuscript.

We have added a benchmark to use matOptimize (Ye et al., Bioinformatics 2022; developed by UShER team) to refine the topology of the inserted trees from TIPars, UShER and MAPLE in SARS2-100k dataset (Figure 4). matOptimize utilizes fast SPR moves. After topology refinement, the refined trees had significant smaller parsimony scores but larger RF distance for trees of TIPars and UShER (Figure 4A to 4C). The inserted trees from TIPars had larger LKs than those of being refined in insert10000 and insert20000 and comparatively similar LK values were observed in insert50000 (Figure 4B). A possible reason is that matOptimize is fully parsimony-based method without a promise on the tree log-likelihood. Our provided reference tree in SARS2-100k dataset was built by a maximum likelihood method (IQTREE2), so the RF distance after refinement by matOptimize may not become smaller. The refined trees from MAPLE had smaller RF distances, larger LKs and smaller parsimony scores compared to those of without refinement which suggests a tree refinement can be useful for the inserted tree of MAPLE.

In the PANGOlins analysis, how accurate are the pangolin lineages assigned to tips in the full tree? The accuracy of the lineage designations is assumed correct, but this might not be the case. PANGO-lineages have been defined using different trees overtime (first iqtree and later USHER).If the full iqtree built here is better than the one used during designation the correct placement may lead to the wrong pango lineage.

**Authors’ reply:** To the best of our understanding, there is no published matric to evaluate the accuracy of PANGO lineages assigned to all tips in a tree because of two agnostic reasons: wrong label by PANGO nomenclature and wrong topology of the tree. Instead of comparing the PANGO lineage labels of all queries, we only consider those queries placed in subtrees of which PANGO lineage labels were annotated by its descendant reference taxa if all of them were monophyletic. This filtering may help to consider only queries with correct lineage labels from PANGO nomenclature to a certain degree.

Methods 330: The wording here is a little unclear. It seems like the number of sites that differ between Q, A and P is summed not the number of mutations since the example given would require at least 2 mutations.

**Authors’ reply:** TIPars considers the mutation of query Q (ACG**T**) differ to **BOTH** of node A (ACC**G**) and node B (ACG**C**) which would lead to the pendant length in Figure 1A. At the 4^th^ site, the genetic character **‘T’** in Q differs to both characters ‘**G’** in node A and ‘**C’** in node B. But at 3^th^ site of Q, though the character **‘G’** differs to characters ‘**C’** in node A, it is the same as ‘**G’** in node B which will not be counted.

Line 342: What is sigma in the equation for l_{P-Q}?

**Authors’ reply:** $\sigma$ is the minimum substitution score that was firstly described at Line 376 and we have also added its definition after this equation at Line 389 of the revised manuscript.
