# Supplementary material for: Robust expansion of phylogeny for fast-growing genome sequence data
Source: PLoS Comput Biol. 2024 Feb 8;20(2):e1011871. doi: 10.1371/journal.pcbi.1011871 (PMC10898724; doi:10.1371/journal.pcbi.1011871)
Supplement: Attachment — Submitted filename: Reply_to_ReviewComments_R2.docx [file pcbi.1011871.s023.docx]

**Authors’ Reply to Review Comments**

Thank you for your extensive revisions in response to the Reviewers' comments and criticism, which have resulted in a substantially improved manuscript nearly ready for acceptance. However, one of the Reviewers still has a few queries that need to be addressed prior to acceptance. I would like to see one last version of this manuscript that addresses these issues. Note, I do not believe a full comparison to UShER-Sampled is needed to justify publication of a TIPars manuscript. That said, this manuscript would still benefit from an explicit acknowledgement of these updated tool and its implications on the findings presented.

Reviewer's Responses to Questions

Comments to the Authors:
Please note here if the review is uploaded as an attachment.

Reviewer #1: The authors have addressed all questions well, and appropriately updated related texts and figures.

Reviewer #2: The revised manuscript shows significant improvement and the authors have successfully addressed most of my previous concerns. However, there are still a few unresolved issues and therefore, I recommend a minor revision.

1. The authors are incorrect in stating that UShER and MAPLE also require an MSA for phylogeny expansion as those tools are reference-based, and therefore, they only require pairwise alignments of new sequences to a single reference sequence. Because inferring an MSA is significantly more expensive than inferring a set of pairwise alignments to a reference, I feel this is a serious limitation of TlPars currently.

**Authors’ reply:** We are sorry for the confusion. In fact, the MSAs of SARS-CoV-2 genomes (including query samples) input for TIPars, UShER and MAPLE are the same and downloaded from GISAID though their file formats may be different. These MSAs are generated by a set of pairwise alignments to a reference by GISAID. We have deleted this statement in the discussion of the revised manuscript.

2. The authors did not incorporate my suggestion to evaluate usher-sampled. This is a new program with several optimizations which have been recently described in Hinrichs et al. Nature Genetics 2023.

**Authors’ reply:** We appreciate the contribution of UShER tool suite (including the latest update to UShER-sampled) for maintaining the huge SARS-CoV-2 phylogenetic tree with millions of genome sequences in near real time. UShER is the fastest using the smallest memory among our tested tools (Table 1) and the absolute difference would become larger when dealing more query samples (S2 Table). In contrast, TIPars performed more robust in phylogenies with both similar and divergent sequences than UShER (Fig. 1 and Fig. 3) with feasible runtime and memory usage (S2 Table). We have added this acknowledgement at Lines 375-380 in the discussion of the revised manuscript.

3. The authors did not satisfactorily answer my question about the scalability of TlPars. Even though a fair comparison with millions of SARS-CoV-2 sequences may not be possible, it would be helpful to understand if TlPars can handle those many sequences.

**Authors’ reply:** Thank you for the suggestion to evaluate the scalability of TIPars. We have done additional tests to add 10k, 20k, 50k, 100k, 200k novel genome sequences into the SARS2-100k reference tree (S2 Table). TIPars took less than one day (21 hours and 24 minutes) to insert 200k SARS-CoV-2 genome sequences into the 100k-taxa reference tree while that of UShER was 9 hours and 35 minutes. We have added this result at Lines 109-111 of the revised manuscript.

**S2 Table. Runtime and memory usage for multiple taxa insertion to the 100k-taxa reference tree.**

| Number of  added samples | Runtime (HH:MM:SS) | | Peak memory usage (GB) | |
| --- | --- | --- | --- | --- |
|  | **TIPars** | **UShER** | **TIPars** | **UShER** |
| 10k | 0:22:37 | 0:10:54 | 3.10 | 0.22 |
| 20k | 0:50:47 | 0:21:10 | 3.44 | 0.42 |
| 50k | 2:41:17 | 1:10:58 | 4.50 | 1.39 |
| 100k | 7:25:55 | 3:24:34 | 10.76 | 3.69 |
| 200k | 21:23:51 | 9:35:10 | 17.49 | 6.72 |

Tests were run on a server with 32 Intel Xeon Gold 6242 CPU cores for 10 repeated runs.

4. In Fig. 4C, can the authors explain why the parsimony score is decreasing when more sequences are being added? I think this should not happen.

**Authors’ reply:** The benchmark in Fig. 4 for the tree refinement is based on leave-many-out test that we removed a set of taxa from the reference tree and reinserted them back to the reduced tree. The resulting tree and the original reference tree are both containing the same number of taxa, 100k taxa in our test. The decreasing of parsimony score means the method did improve the tree. That is described at Lines 268-271 of the revised manuscript.

Reviewer #3: I appreciate the time and effort the authors have taken to address mine and the other reviewers' comments. I have no remaining concerns with the manuscript.
